# Challenges to the management of curable sexually transmitted infections

**DOI:** 10.1186/s12879-015-1061-2

**Published:** 2015-12-01

**Authors:** Marcus Y Chen, Sepehr N Tabrizi

**Affiliations:** Melbourne Sexual Health Centre, Alfred Health, Carlton, VIC 3053 Australia; Central Clinical School, Monash University, Clayton, VIC 3800 Australia; Department of Obstetrics and Gynaecology, University of Melbourne, Melbourne, Australia; Department of Microbiology and Infectious Diseases, Royal Women’s Hospital, Melbourne, Australia; Murdoch Children’s Research Institute, Melbourne, Australia

## Abstract

Each year, hundreds of millions of new cases of curable sexually transmitted infections (STIs) occur worldwide resulting in reproductive and other serious sequelae, as well as enhanced transmission of HIV. The clinical management and control of these STIs should include as a minimum access to services that provide timely and accurate diagnostic testing together with effective treatment. The provision of appropriate treatment is challenged by the development of increasing antimicrobial resistance, in particular with gonorrhoea and *Mycoplasma genitalium* infections, requiring new treatments and management algorithms. In addition, infections such as chlamydia, syphilis and trichomoniasis, which show few signs of resistance, are nevertheless highly prevalent and require better public health control measures. While these may be achievable in high income countries, they are still beyond the reach of many low and middle income countries, making substantial improvements in STI management and reductions in STI prevalence challenging.

## Editorial

In 2008, the World Health Organization (WHO) estimated that globally there were 499 million new cases of curable sexually transmitted infections (STIs), including gonorrhoea, chlamydia, syphilis, and trichomoniasis, occurring every year. Discouragingly, this was even higher than the estimated 448 million cases reported in 2005 [1]. Each of these cases, mostly detected in young men and women, represents a person diagnosed with what is generally a stigmatizing condition with potentially serious reproductive or other sequelae. These include ectopic pregnancy, pelvic inflammatory disease, preterm birth, female infertility, neonatal death, and enhanced acquisition and transmission of HIV, just to name a few [1, 2]. This begs the question, what more can be done to improve the diagnosis, treatment and control these curable infections? This special issue of *BMC Infectious Diseases* focuses on the challenges confronting the clinical management of several key treatable STI and STI related syndromes with experts in the field offering opinions on where future efforts should be focused. These include infections where antimicrobial resistance is established and increasing as well as infections that show few signs of resistance but are nevertheless highly prevalent with the need for better control.

Over several decades, *Neisseria gonorrhoeae* has successively developed antimicrobial resistance to multiple classes of antibiotics and has been identified by the United States Center for Disease Control as one of the top urgent antibiotic resistance threats [3]. Case reports of high level resistance to ceftriaxone have rung alarm bells and further stimulated efforts towards the discovery of new alternative treatments [4, 5]. In this issue Unemo reviews these and other cases, highlights the need for continued vigilance, and discusses potential new agents in the development pipeline aimed at combatting this mercurial bacterium [6].

*Mycoplasma genitalium* is a common cause of male urethritis and is found in women with pelvic inflammatory disease [7]. Like gonorrhoea, *Mycoplasma genitalium* has developed increasing resistance to various antibiotic classes, a problem that is currently under recognized and in pressing need for greater attention. Jensen *et al.* provide a detailed overview of data pointing to the growing antimicrobial resistance evident with *M. genitalium* infections [8]. It is difficult to envisage any reversal in these trends without the introduction of fundamental measures such as widespread routine diagnostic testing for *M. genitalium*, availability of testing for antimicrobial resistance, and ongoing surveillance. In many high income countries these are in place for gonorrhoea but notably absent for *M. genitalium.*

Moi *et al.* explore the treatment implications of *M. genitalium* resistance further in their proposed guidance on the clinical management of men presenting with non-gonococcal urethritis, one of the most common STI related syndromes [9]. This currently presents a major clinical challenge as most urethral chlamydia will clear with azithromycin or doxycycline but both of these antibiotics are becoming increasingly ineffective at eliminating *M. genitalium* urethritis. Moi *et al.* float the idea of withholding treatment from men presenting with non-gonococcal urethritis until a diagnosis of chlamydia or *M. genitalium* is confirmed by laboratory testing. This concept is worthy of further exploration and may need to be tailored to different clinical settings. Better drugs and more rational management algorithms for non-gonococcal urethritis are needed.

While antimicrobial resistance to chlamydia is fortunately not currently contributing to the treatment failures to any significant extent, there continues to be uncertainty over whether single dose azithromycin, which has been the mainstay of treatment for uncomplicated genital chlamydial infections for many years, or doxycycline, should be the preferred treatment for chlamydia [10]. The question of whether doxycycline should replace single dose azithromycin as first line therapy for non-gonococcal urethritis in men based on efficacy against chlamydia and possible induction of macrolide resistant *M. genitalium* warrants further investigation [10]. A growing number of observational studies have suggested that azithromycin is inferior to doxycycline for the treatment of rectal chlamydia [11]. Kong *et al.* delve into these issues in their review of chlamydia treatment and call for randomized controlled trials to definitively determine if doxycycline should replace single dose azithromycin for the treatment of rectal chlamydia [12]. Several studies have pointed to rectal chlamydia being prevalent among not only men who have sex with men, but also women [13].

Globally, syphilis remains a pressing concern with international data indicating a resurgence of syphilis among men who have sex with men and unacceptably high rates of congenital syphilis [14, 15]. To highlight the magnitude of this, the WHO estimated that in 2008 syphilis infections in pregnancy led to 305,000 fetal and neonatal deaths and 215,000 infants at risk of dying from prematurity, low birth weight or congenital syphilis worldwide [1]. Syphilis also drives acquisition of HIV infections, worrying as both syphilis and HIV are overrepresented in many populations of MSM [16]. In their review on the challenges in the management of adult syphilis, Tuddenham *et al.* revisit whether standard treatments for syphilis are adequate in HIV positive patients [17]. They also discuss uncertainty over the management of patients who remain serofast following syphilis treatment and developments in laboratory diagnostics.

Given the extraordinarily high global prevalence of *Trichomonas vaginalis* and its potential to promote HIV transmission, further investment into the optimal diagnosis, treatment, and control of *T. vaginalis* is clearly needed. Kissinger provides a comprehensive review on the epidemiology, diagnosis and management of this infection [18]*.* The fact that *T. vaginalis* screening of HIV positive women in the US alone would save an estimated $160 million in lifetime costs from new HIV infections prevented underscores the public health importance of the optimal detection and management of this protozoal infection [19].

While not usually regarded as a curable STI, bacterial vaginosis is common among women presenting with vaginal discharge and linked to reproductive sequelae and enhanced HIV transmission [20, 21]. Bradshaw *et al.* discuss emerging areas for research into bacterial vaginosis aimed at reducing recurrence of this condition which is common following treatment [22]. These include studies to help elucidate the postulated role of reinfection from sexual partners, vaginal microbiota, and vaginal biofilm in the pathogenesis of bacterial vaginosis and possible interventions targeting these.

The WHO global strategy for the prevention and control of STI stipulates that comprehensive STI management should include as a minimum, accurate diagnosis by syndrome or laboratory diagnosis, plus effective treatment to prevent complications and further transmission [23]. While laboratory testing is taken for granted in high income countries, they are still beyond the reach of many low and middle income countries. Better performing point of care tests and use of self-collected sampling for several STI bring some hope of more access to diagnostic testing. Similarly, while reasonable adherence by health providers to local treatment guidelines governing antibiotic use for STI should be expected in well-resourced countries, less regulated use of antibiotics in resource limited settings threatens to only compound antimicrobial resistance. Stark differences in levels of health funding, health service infrastructure, together with what is often a low priority for STI on the political agenda, means meaningful improvements in STI management with reductions in STI prevalence will for the foreseeable future remain challenging.
